# Bio-mining of Lanthanides from Red Mud by Green Microalgae

**DOI:** 10.3390/molecules24071356

**Published:** 2019-04-06

**Authors:** Mária Čížková, Dana Mezricky, Marian Rucki, Tivadar M. Tóth, Vít Náhlík, Vojtěch Lanta, Kateřina Bišová, Vilém Zachleder, Milada Vítová

**Affiliations:** 1 Laboratory of Cell Cycle of Algae, Centre Algatech, Institute of Microbiology, Czech Academy of Sciences, Novohradská 237, 379 81 Třeboň, Czech Republic; cizkova@alga.cz (M.Č.); nahlik@alga.cz (V.N.); vlanta@centrum.cz (V.L.); bisova@alga.cz (K.B.); zachleder@alga.cz (V.Z.); 2 Institute of Medical and Pharmaceutical Biotechnology, IMC FH Krems, Piaristengasse 1, A-3500 Krems, Austria; dana.mezricky@fh-krems.ac.at; 3 Laboratory of Predictive Toxicology, National Institute of Public Health, Šrobárová 48, 100 42 Prague, Czech Republic; ruckim@yahoo.com; 4 Department of Mineralogy Geochemistry and Petrology, University of Szeged, Egyetem u. 2, H-6722 Szeged, Hungary; mtoth@geo.u-szeged.hu; 5 Faculty of Science, University of South Bohemia, Branišovská 1760, 370 05 České Budějovice, Czech Republic; 6 Department of Functional Ecology, Institute of Botany, Czech Academy of Sciences, Dukelská 135, 379 81 Třeboň, Czech Republic

**Keywords:** microalgae, lanthanides, red mud, bio-mining, recovery, toxicity

## Abstract

Red mud is a by-product of alumina production containing lanthanides. Growth of green microalgae on red mud and the intracellular accumulation of lanthanides was tested. The best growing species was *Desmodesmus quadricauda* (2.71 cell number doublings/day), which accumulated lanthanides to the highest level (27.3 mg/kg/day), if compared with *Chlamydomonas reinhardtii* and *Parachlorella* *kessleri* (2.50, 2.37 cell number doublings and 24.5, 12.5 mg/kg per day, respectively). With increasing concentrations of red mud, the growth rate decreased (2.71, 2.62, 2.43 cell number doublings/day) due to increased shadowing of cells by undissolved red mud particles. The accumulated lanthanide content, however, increased in the most efficient alga *Desmodesmus quadricauda* within 2 days from zero in red-mud free culture to 12.4, 39.0, 54.5 mg/kg of dry mass at red mud concentrations of 0.03, 0.05 and 0.1%, respectively. Red mud alleviated the metal starvation caused by cultivation in incomplete nutrient medium without added microelements. Moreover, the proportion of lanthanides in algae grown in red mud were about 250, 138, 117% higher than in culture grown in complete nutrient medium at red mud concentrations of 0.03, 0.05, 0.1%. Thus, green algae are prospective vehicles for bio-mining or bio-leaching of lanthanides from red mud.

## 1. Introduction

The group of rare earth elements (REEs) includes scandium (Sc), yttrium (Y), and a series of 15 other elements from the lanthanide series. Although they differ in atomic number (21 for Sc, 39 for Y and the others from 57 to 71), they exhibit similar physical and chemical properties [1]. In general, lanthanides can be divided into light (from lanthanum to europium) and heavy REEs (from gadolinium to lutetium, including Y and Sc). Due to their unique magnetic and catalytic properties, lanthanides are widely used in almost all electronic and clean energy technologies. As a consequence of the development of modern technologies, the demand and prices for lanthanides are steadily increasing, leading to potential exhaustion of current limited supplies. Lanthanides are therefore critical raw materials because of their high supply risk and above average economic importance in comparison with other raw materials [2].

The risk of reduced availability of resources in China (the main world producer), and possibly of a depletion of other natural resources, raises the need for more efficient and sustainable (bio)mining processes, even from low grade ores, as well as efficient methods for recycling of lanthanides, e.g., from waste material. Although recycling is far from reaching its full potential, it has some advantages such as a lack of radioactive impurities and an economic independence of supply from primary sources.

Another potential, so far very little exploited, source of lanthanides may be found in so called red mud. Red mud is a by-product of the production of alumina (aluminum oxide) from bauxite ore during the standard Bayer process. Red mud is currently being produced at a global rate of 150 million tons annually [3], while a cumulative amount of 4 billion tons of bauxite residue have already been stored worldwide since 2015 [4]. However, less than 2% of the residue produced annually is currently being reused [5], due to difficulties related to high pH, salinity, low solid content, size of fine particles and leaching of metals [6]. Information on the recovery of valuable metals (reviewed in [7]) provides an insight into the full potential of red mud as an economic resource rather than a waste material.

Red mud has been deposited in large dumps for decades, where its chemical and mineralogical composition may change significantly due to interactions with groundwater. As a result, red mud consists of a wide spectrum of minerals, some of which still reflect the source sedimentary rock, bauxite, while others have developed due to chemical treatment under industrial conditions and some mineral phases formed in the deposit ponds.

During the Bayer process, bauxite ore is heated, along with a sodium hydroxide solution, at a temperature of 150 to 200 °C to obtain soluble aluminum (oxy) hydroxides. After separating the aqueous solution of aluminum hydroxide from solid impurities, the remaining solid fraction is called bauxite residue, while the waste sludge is called red mud [8]. The residue of the chemical treatment is still very high in sodium and has a pH > 11. Among the minor components of red mud, lanthanides and scandium are of special interest since, based on recent research, extraction of these components from red mud is a real possibility (e.g., [9]).

Methods for extraction of lanthanides from ores, such as pyrometallurgy and hydrometallurgy have severe negative environmental impacts, as well as being expensive. Currently, industrial extraction of lanthanides from monazite involves either a basic process that uses concentrated sodium hydroxide or an acidic process that uses concentrated sulfuric acid. These processes generate large amounts of hazardous waste containing thorium and uranium [10]. Bio-mining techniques are generally less energy-intensive and less polluting.

Research has therefore recently focused on more environmentally-friendly technologies of metal recovery from secondary resources [11]. Biological methods might offer an alternative to physicochemical recycling techniques, although they are still limited by high costs and low element selectivity [12]. Microbial biotechnologies such as bio-mining, bio-leaching, bio-recovery, bio-sorption, bio-electrochemical recovery, bio-precipitation, bio-flotation, bio-reduction or bio-accumulation that can be used in the recovery of critical and scarce metals are explained in detail and summarized, for example, in [12,13,14,15,16]. Considerable progress has been achieved in the development of efficient biological methods for lanthanide recovery using microorganisms such as bacteria, fungi, cyanobacteria or algae [17,18].

An important aspect of any efficient bio-recovery method is selection of a suitable species. Different microorganisms were investigated for exploitation in scarce metal recovery biotechnology. Filamentous fungi *Penicillium tricolor* and *Aspergillus niger* producing citric acid and oxalic acid were used for bio-leaching of lanthanides from red mud [19,20]. In the case of the red alga *Galdieria sulphuraria*, interactions of phosphoproteins and Ca-binding proteins with lanthanides were identified as factors in the bio-sorption of lanthanides [17]. Park and colleagues reported the expression of lanthanide-binding tags on the surface of *Caulobacter crescentus,* leading to the adsorption of lanthanides [18]. The only selective bio-accumulation so far was described in the fungus *Penidiella* sp. T9. This fungus selectively accumulated dysprosium from acidic solutions [21].

Only a few studies of lanthanide recovery by algae or cyanobacteria have been published. With the exception of the red alga *Galdieria sulphuraria* [17], the live macroalga *Gracillaria gracilis* was effectively used to recover lanthanides from waste water [22]. Dried or carbonized biomass of the green alga *Parachlorella* was used for bio-sorption and reversible desorption of lanthanides from aqueous solution [23]. Studies of bio-remediation of red mud were performed with the cyanobacterial species *Phormidium* and *Oscillatoria* [24]. Results indicated that these microorganisms were able to reach a high growth rate in the presence of red mud-supplemented nutrient medium. Several studies have shown that lanthanides accumulate in chloroplasts [25,26,27,28]. It was demonstrated that selective deposition of individual lanthanides in chloroplasts or the cytoplasm occurs in the green alga *Desmodesmus quadricauda*. Nd and Ce were located in the chloroplast while La and Gd were found in the cytoplasm [29]. The advantage of using phototrophic organisms for remediation is the sequestration of CO_2_ during their growth and assimilation of pollutants present in the waste-water. Moreover, the biomass produced can be further reused for feed, food or fertilizers [30].

The aim of this study was to examine the ability of selected species of green microalgae to grow in the presence of red mud and to accumulate lanthanides from this lanthanide-rich material. In order to examine bio-absorption capacity and physiological effects of lanthanides, the three species *Desmodesmus quadricauda*, *Chlamydomonas reinhardtii,* and *Parachlorella kessleri* were cultivated in the presence of different concentrations of red mud. As a comprehensive determination of the content of lanthanides accumulated in algal biomass, inductively coupled plasma mass spectrometry was used. The simultaneous verification of accumulation and the localization of lanthanides were examined using fluorescence microscopy. The work describes the potential of green algae for bio-mining of lanthanides from red mud or bio-leaching.

## 2. Results

### 2.1. Composition of Lanthanides and Other Metals in Red Mud

To consider the extensive waste red mud deposits as a potential source for bio-mining lanthanides, the composition of these elements in different locations and depths of the mud disposal site had to be analyzed. For experiments, samples were collected at a depth of approximately 1–1.2 m measured from the red mud surface. At this depth, the state of the red mud was gelatinous and wet. From the list of lanthanides analyzed (Table 1), cerium, lanthanum and neodymium were found to be proportionally the most abundant at 36.5, 17.2, and 14.7% respectively, i.e., representing 68.4% of the total amount of lanthanides. 

High proportions of the heavy REEs scandium and yttrium, 8.2 and 10.1% respectively, were also found. The proportion of other lanthanides were relatively low and ranged from 3.2 to 0.2% (Table 1).

Analyses of other elements (Table 2) demonstrated their presence at very high levels, about three orders of magnitude higher (g/kg) than lanthanides (mg/kg). The red color of the mud derives from compounds of the most frequent element, iron (53% of the total content of metal elements, Table 2). Most of these elements, particularly microelements, could be used as inorganic compounds for the growth of algal cultures (see the composition section in Methods). 

### 2.2. Selection of a Suitable Model Organism for Cultivation with Red Mud

To investigate the bio-mining of lanthanides from red mud by algae, initial experiments were carried out to select the appropriate algal species for their ability to grow in the presence of red mud and accumulate lanthanides from it. The three species of green algae *Desmodesmus quadricauda*, *Chlamydomonas reinhardtii* and *Parachlorella kessleri* were selected.

**Figure 1:**
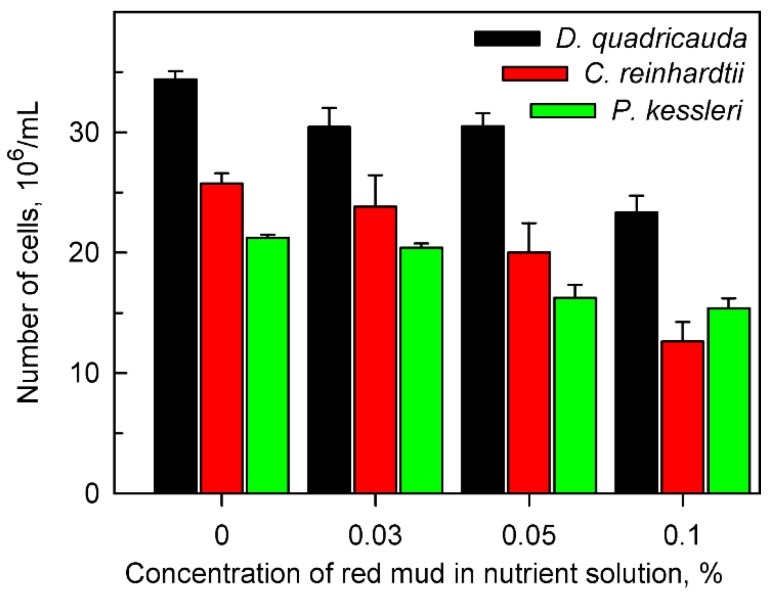
Cell number of *Desmodesmus quadricauda*, *Chlamydomonas reinhardtii* and *Parachlorella kessleri* after 48 h of growth in the absence (0%) or presence of different concentrations (0.03, 0.05, 0.1%) of red mud in nutrient medium suitable for the given species. All the cultures were diluted to the same initial number of cells (8 × 10^5^/mL) at the beginning of each experiment.

A stock 10% suspension of red mud in water (*w/v*) was used for all experiments. Aliquots of this stock suspension were added to corresponding algal nutrient media (see Methods) to final concentrations of 0.03, 0.05 and 0.1% (*w/v*). The control culture was grown in the absence of red mud in a nutrient medium (Figure 1). The final cell number was estimated after 48 h of growth at 30 °C, and an incident light intensity of 500 μmol/m^2^/s. All experimental cultures started growth at a cell concentration of 8 × 10^5^/mL.

Particles of red mud suspended in nutrient medium were only partially solubilized and with increasing amounts of added suspension, the insolubilized particle content increased. Shadowing of cells by insoluble particles of red mud caused a decrease in the mean light intensity (light intensity experienced by cells, for determination see Material and Methods). The measured mean light intensities in cultures grown at concentrations of 0, 0.03, 0.05 and 0.1% red mud were 500, 400, 200 and 100 μmol/m^2^/s, respectively. The decrease in mean light intensity with increasing levels of red mud caused slower growth of cell cultures for all species tested (Figure 1, Table 3). Nevertheless, for any concentration of red mud, *D. quadricauda* grew better than the other two algal species (Figure 1, Table 3).

**Figure 2:**
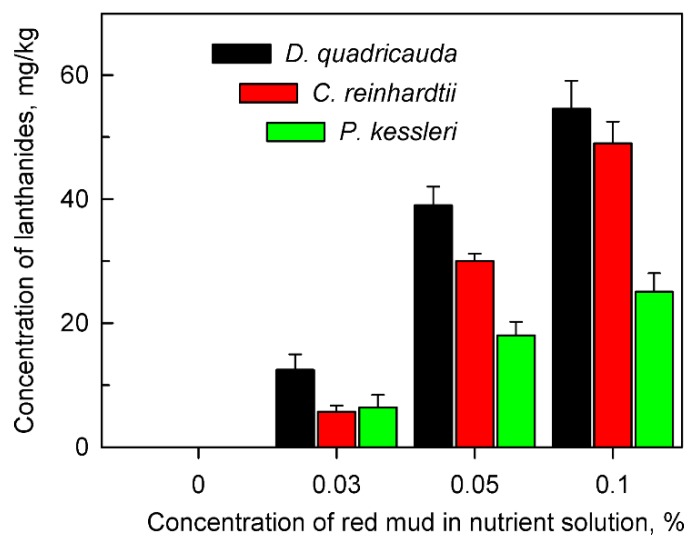
Total amount of lanthanides accumulated in cells of *Desmodesmus quadricauda*, *Chlamydomonas reinhardtii* and *Parachlorella kessleri* after 48 h of growth in the absence (0%) or presence of different concentrations (0.03, 0.05, 0.1%) of red mud in nutrient medium suitable for the given species. No lanthanides were found in cells grown in the absence of red mud.

The cells of all algal species tested were able to accumulate lanthanides intracellularly from the red mud suspension (Figure 2). The concentration of bio-absorbed lanthanides increased with increasing concentrations of red mud in the nutrient medium. In spite of the fact that cells in the presence of high concentrations of red mud (0.1%) grew much slower than those at lower concentrations, their intracellular content of lanthanides was much higher (Figure 2). The alga *D. quadricauda* accumulated more lanthanides in comparison with *C. reinhardtii* and *P. kessleri* (Figure 2).

To find the most appropriate species for more detailed experiments, the proportional bio-absorption of three most frequent lanthanides, cerium, lanthanum and neodymium from red mud (36.5, 17.2 and 14.7% of total lanthanides, respectively, see Table 1) was followed in all species, at concentrations of red mud (0.1% and 0.03%), the level of which was assumed to be sufficient for this purpose. 

**Figure 3:**
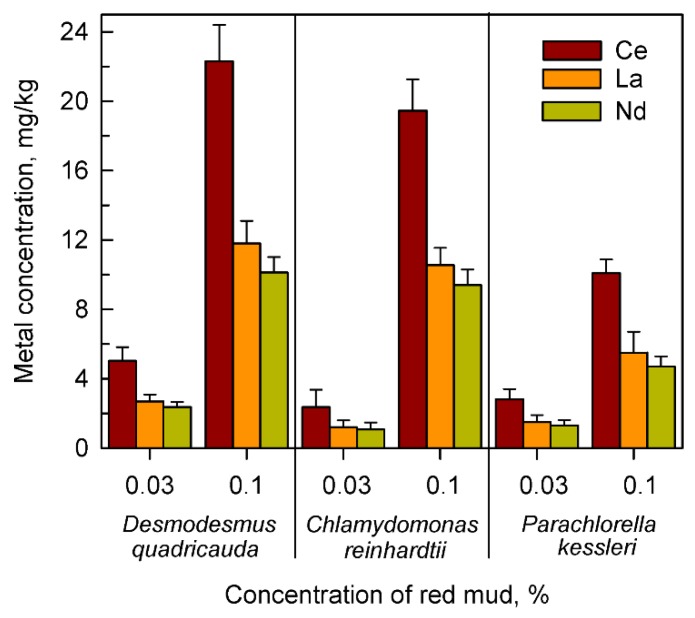
The content of cerium, lanthanum and neodymium in cells of *Desmodesmus quadricauda*, *Chlamydomonas reinhardtii* and *Parachlorella kessleri* grown in the presence of different concentrations (0.03, 0.1%) of red mud in complete nutrient medium suitable for the given species.

It was confirmed that the highest levels of all three lanthanides, at both lowest and highest concentrations of red mud were found in *Desmodesmus quadricauda* (Figure 3). Higher levels of lanthanides accumulated with higher concentrations of red mud. Lanthanides accumulated in about the same proportion as they were present in an extracellular suspension of red mud (compare data in Table 1 and in Figure 3).

### 2.3. Cultivation of Desmodesmus quadricauda with Red Mud in Incomplete Nutrient Medium 

Analyses of red mud for the content of different metal elements (Table 1 and Table 2) provided evidence for the presence of many metals that are also present in algal nutrient media (see Materials and Methods). From the comparative list of red mud elements and the elements used in nutrient medium, it is apparent that elements B, Mn, Co, Ni, Cu, Zn are essential microelements for algal growth (compare Table 2 and Table 4, bottom part). 

To substitute these metals in a nutrient medium with those present in red mud could decrease the cost and simplify the preparation of these media, particularly in scale-up photo-bioreactors for biotechnological applications, as well as to improve the bio-leaching of red mud waste. 

**Figure 4:**
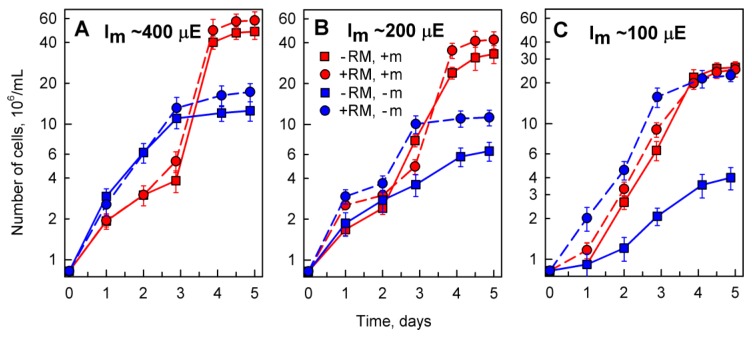
Changes in number of *Desmodesmus quadricauda* cells grown in the absence (-RM) or presence (+RM) of different red mud concentrations in different nutrient media: 0.03% (panel **A**), 0.05% (panel **B**), 0.1% (panel **C**). The experimental variants were grown either in complete (+m) or in incomplete nutrient media (−m) lacking added microelements. Mean light intensity (I_m_) decreased due to increasing shadowing by solid particles with increasing concentration of red mud: 400 µE (panel **A**), 200 µE (panel **B**), 100 µE (panel **C**). Mean light intensity of the control cultures (−RM) was adjusted so it matched the mean light intensities of the corresponding red mud treated culture.

**Figure 5:**
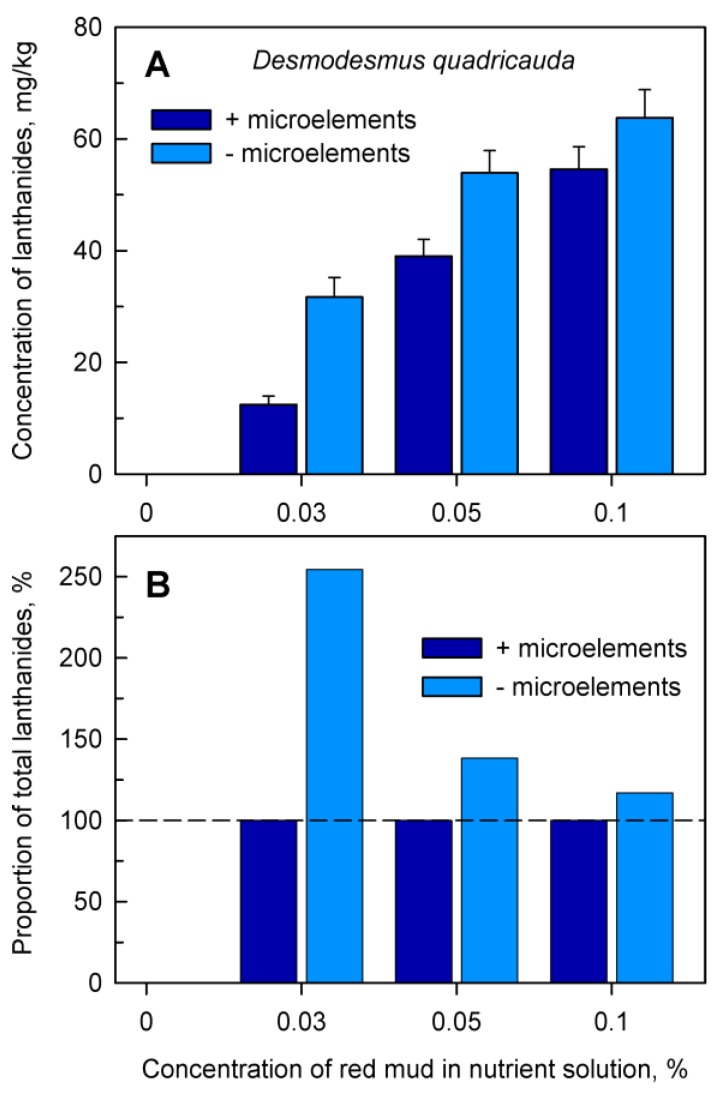
Total amount of accumulated lanthanides in *Desmodesmus quadricauda* cells (**Panel A**) after 48 h of growth in the absence (0%) or in the presence of different concentrations (0.03, 0.05, 0.1%) of red mud, either in complete nutrient medium (+microelements) or in medium without added microelements (−microelements). **Panel B**: Proportion of total lanthanides in cells grown in nutrient medium without added microelements normalized to the content of lanthanides in cells grown in complete nutrient medium (set to 100%, highlighted by dashed horizontal line).

**Figure 6:**
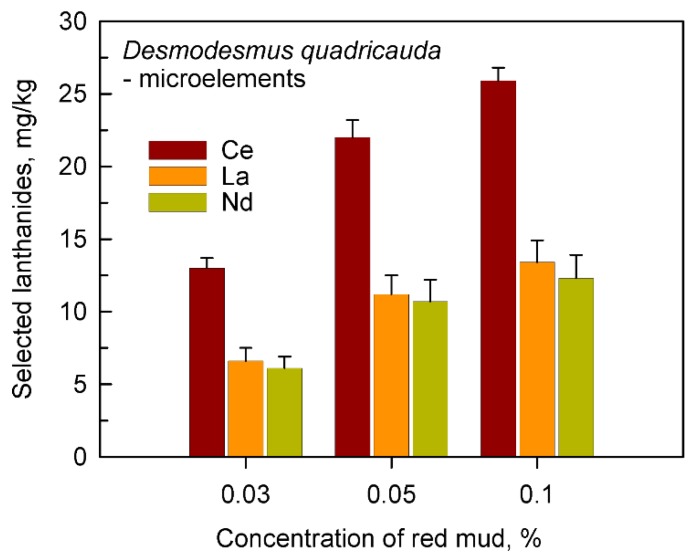
The content of cerium, lanthanum and neodymium in cells of *Desmodesmus quadricauda* grown in incomplete nutrient medium lacking added microelements in the presence of 0.03, 0.05 and 0.1% (*w/v*) of red mud.

The alga *D*. *quadricauda* was selected for these experiments because, from algal species tested, it had the best growth and the highest bio-sorption of lanthanides. Algal cultures grown either in complete medium containing microelements (+m) or in incomplete medium lacking added microelements (−m) (for the composition of the media see Material and Methods), were treated with red mud at concentrations of 0.03, 0.05 and 0.1% (*w/v*). Control cultures were grown in the same medium in the absence of red mud (0%) (Figure 4, Figure 5 and Figure 6). The incident light intensity (I_i_) used for all red mud-treated cultures was about 500 µE (μmol/m^2^/s), but the corresponding mean light intensity (I_m_) (see Materials and Methods for measurement and calculation) decreased with increasing concentrations of red mud due to shadowing effect of undissolved particles. Thus, I_m_ was about 400, 200 and 100 µE for concentrations of 0.03, 0.05 and 0.1%, respectively. To enable a comparison with growth of control cultures, the cultures were grown separately at the same mean light intensities as for algae grown in the corresponding red mud concentrations (Figure 4).

Growth of algal cultures was followed as changes in cell number over a 5 day period. During this period, the cultures underwent lag and exponential phases of growth and entered into the stationary phase of growth. The stationary phase was reached already after 3 days in culture grown in incomplete medium (−m) (Figure 4, blue lines) and after 4 days of growth in complete nutrient medium (+m) (Figure 4, red lines). 

The findings illustrated in Figure 4 revealed that the cells not only could grow in red mud in incomplete nutrient medium without added microelements but they grew better than in control red mud-free medium (Figure 4A,B). This effect was most pronounced at low mean light intensity at high concentrations of red mud (Figure 4C) suggesting that the presence of red mud is advantageous for the full supply of elements missing from the nutrient medium. Such cultures grew to the same cell concentrations (Figure 4C, blue circles) as the cultures grown in complete nutrient medium, either in the absence (Figure 4C, red squares) or at a high concentration of red mud (Figure 4C, red circles). 

Analyses of the accumulation of total lanthanides in cells provided evidence that at any concentration of red mud, cultures grown in nutrient medium lacking added microelements accumulated significantly higher levels of lanthanides (Figure 5, light blue columns) than those grown in complete nutrient medium. (Figure 5, dark blue columns). When the lanthanide content in the cells grown in complete medium in the presence of microelements was set as 100% (Figure 5B, dark blue columns), the proportion of lanthanides decreased from 250% to 117% of the control value with increasing concentrations of red mud (Figure 5B, light blue columns). 

The same was also confirmed in more detailed analyses for the most frequently found individual lanthanides Ce, La, and Nd (Figure 6). The absolute level of Ce, La and Nd in cultures grown in the absence of added microelements and in the presence of any concentration of red mud was higher than in cells grown in complete nutrient medium containing microelements. This is well illustrated when comparing cells grown in complete medium in the presence of microelements from Figure 3 or in the absence of added microelements (Figure 6).

### 2.4. Localization of Lanthanides in Algal Cells

To verify that lanthanides from red mud found in algal biomass by ICP-MS had accumulated intracellularly and were not simply adsorbed to cell walls, the cells of three experimental algal species were stained with the fluorescent dye Fluo-4 (see Materials and Methods). 

**Figure 7:**
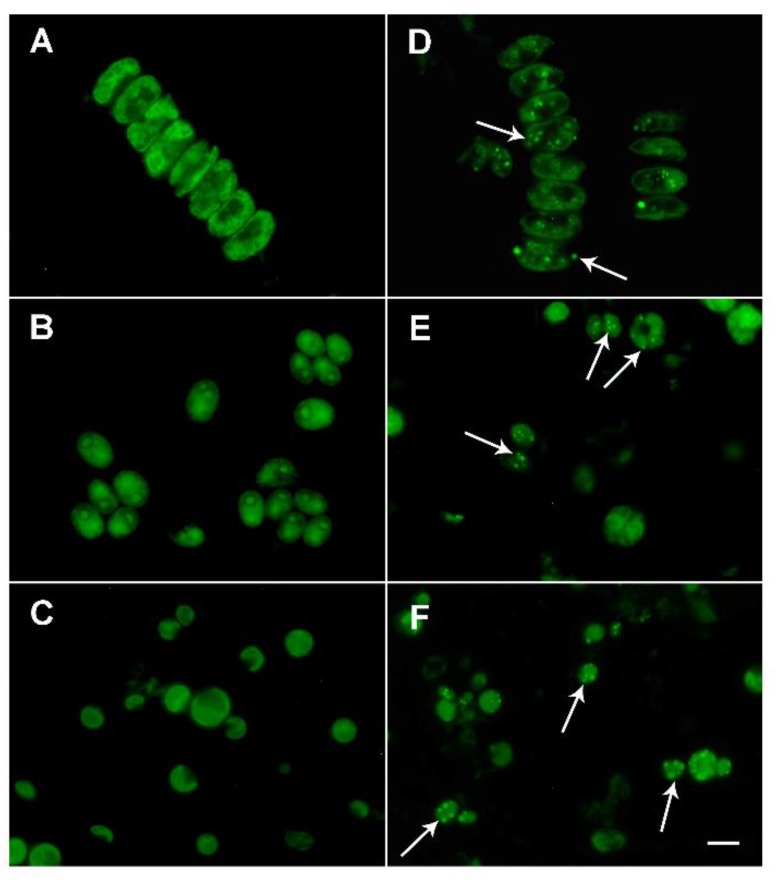
Fluorescence microphotographs after Fluo-4 staining of *Desmodesmus quadricauda* (**A**,**D**), *Chlamydomonas reinhardtii* (**B**,**E**) and *Parachlorella kessleri* (**C**,**F**) grown in the absence (**A**–**C**) or in the presence (**D**–**F**) of 0.1% red mud. Intracellular localization of lanthanides inside the red mud-treated cell are seen as small bright-green bodies, some of them indicated by arrows. The scale bar is 10 µm.

The increased fluorescence signal is a consequence of lanthanide cations binding to the Fluo-4 dye. Staining showed frequent small bright bodies, presumably containing lanthanides in cells grown in the presence of 0.1% red mud (Figure 7D–F). These bodies were not found in untreated cells (Figure 7A–C). The lanthanides were localized both in chloroplasts and the cytoplasm (see arrows in Figure 7D–F).

## 3. Discussion

### 3.1. Red Mud as a Source of Valuable Metals

Production of red mud has increased, providing an almost inexhaustible resource, but due to its toxicity, it is a burden on the environment. Among red mud contaminants, arsenic (As) is of considerable concern [31]. The concentration of As in our samples collected 1–1.2 m deep in the Almásfüzítő landfill (100 mg/kg) exceeded the standards for Protection of Aquatic Life, where As levels in water are regarded as being toxic at concentrations ≥50 µg/L and sediments are toxic at As levels ≥17 mg/kg [32]. On the other hand, the summed content of lanthanides in red mud was about 170 mg per kg of red mud, from which about 80% form the most industrially important elements, cerium, neodymium and lanthanum (Table 1), implying it could serve as a possible source of lanthanides. Besides lanthanides, red mud could also be a valuable source of other metals, such as iron [33] or microelements, which, in the case of algae, are required for their growth. Thus, red mud appears to be a potential raw material for the recovery of valuable lanthanides and a broad range of minor trace elements and, especially in the case of lanthanides, it seems more appropriate to speak about re-mining rather than re-cycling. 

### 3.2. Bio-Mining of Red Mud by Green Algae

**Figure 8:**
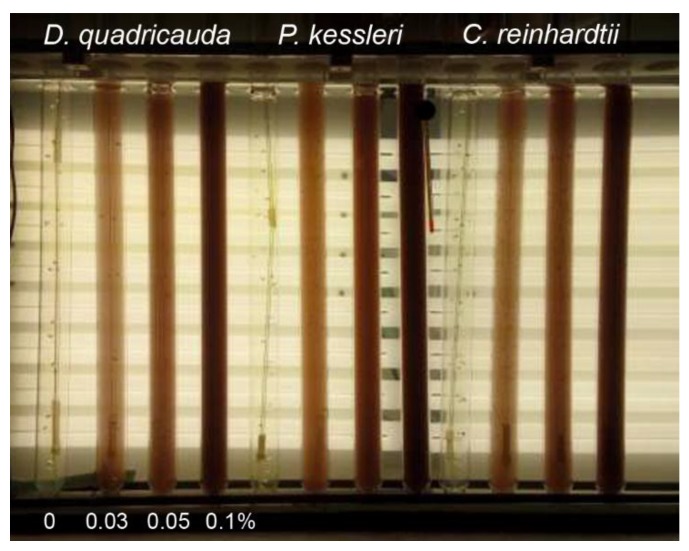
Photobioreactor used for experiments. The photobioreactor consisted of glass cylinders placed in thermostatic water bath illuminated from one side by a panel of dimmable fluorescent lamps. The experimental species cultures were grown either in absence (0%) (control) or in different red mud concentrations (0.03, 0.05, 0.1%).

Our study showed that at least three species of green algae, *D. quadricauda*, *C. reinhardtii* and *P. kessleri*, not only grow but also divide their cells in the harsh red mud environment (Figure 1). All the selected species have been extensively used as model organisms in basic physiological research of algae, cell cycle regulation, molecular biology as well as in biotechnological applications [34,35,36,37] and are well established as rapidly growing and tolerant to possible toxins. Moreover, they were able to efficiently accumulate lanthanides (and other essential metals) from the red mud (Figure 2). Despite different growth rates, all the strains showed a reduction in growth rates in higher red mud concentrations (Figure 1 and Figure 4; Table 3). This could be caused either by red mud toxicity or by light limiting conditions in highly concentrated suspensions of red mud (Figure 8). When the growth conditions were normalized to light intensity (Figure 4), the presence of red mud did not have a toxic but rather a beneficial effect on algal growth. This suggests the reduced growth rates are dictated rather by light conditions and not by red mud toxicity.

The intracellular content of both total (Figure 2) and individual lanthanides (Figure 3) increased with increasing concentrations of red mud. This was valid for all three algal species tested even though efficiencies of lanthanide absorption increased from *P. kessleri* to *C. reinhardtii* and then *D. quadricauda* (see Figure 1, Figure 2 and Figure 3). The three algae accumulated the highest concentration of cerium, followed by lanthanum and neodymium thus reflecting the proportion of lanthanides in red mud (Table 1). At least five-times more lanthanides were accumulated if cultured with 0.1% than with 0.03% red mud (Table 1, Figure 3). This pattern was the same for all taxa, indicating that the mechanism of lanthanide uptake was not species-specific and that the increase in lanthanide accumulation could be driven by higher concentrations of red mud or by a beneficial effect of some red mud component(s) (see below). It has been established that light lanthanides (La, Ce, Pr, Nd) are taken up by organisms preferentially because they are present in the environment at the highest concentrations. This was proven in experiments examining lanthanide concentrations from biota of natural systems, where concentrations within an organism varied with the same pattern (light lanthanides > heavy lanthanides) as observed in surrounding environments [38,39]. Our results clearly prove lanthanide bio-mining capacity of aquatic microalgae but additional investigation is needed to understand the detailed mechanisms behind the bioaccumulation of metals from red mud-containing environments. The differences observed could be caused by many factors such as species-specific growth rates, differing growth optima (red mud concentrations and corresponding differences in illumination), and differences in affinities for lanthanide [40,41]. Previously, the removal of lanthanides from sewage water was demonstrated using algae such as *Chlorella vulgaris*, *Isochrysis galbana* or *Euglena gracilis* [42,43,44]. Another organism with proven ability to concentrate and recover critical rare elements from contaminated water is *Gracilaria gracilis* [44]. In many studies, *Desmodesmus* spp. was used as a bioremediation agent to remove inorganic and organic substances from polluted water [45,46].

The role of photosynthetic algae in the bioremediation of elements from red mud has not been studied in detail. However, several cyanobacteria and fungi have been tested for bio-leaching of bauxite residue [19,24]. Cyanobacteria were found to be less efficient in the re-vegetation of red mud field deposits than fungi because they suffered more under alkaline conditions and a total lack of energy sources. But if the pH was reduced they formed a crust-like structure on the red mud surface [24]. In contrast, fungi were less vulnerable to harsh conditions of red mud, and could even excrete metabolites (such as organic acids, amino acids, and proteins) and form complexes with metal ions. The fungus RM-10 (originally isolated from red mud), was used to extract metals from red mud because of its ability to drastically reduce the pH of the medium supplemented with high concentrations of bauxite residue [19].

For possible future scale-up culturing of algae for bio-mining of lanthanides from red mud, it is important to make sure that the lanthanides are really bio-absorbed into intracellular structures not just adsorbed onto the cell surface. Thus in our study, cells prepared for analysis of the intracellular content of lanthanides (by ICP-MS) were repeatedly washed to remove compounds or metals adsorbed onto the cell surface. Because it cannot be totally excluded that some lanthanides could remain attached to cell walls, even after intensive washing, direct fluorescence microscopic observation was carried out to verify the intracellular accumulation of lanthanides. The Fluo-4 dye is commonly used as a calcium indicator [47] but lanthanides have a higher affinity for the dye molecules, so it can be used to visualize lanthanides. Lanthanides in the cells were concentrated in bodies of different sizes and locations (Figure 7). Some were inside chloroplasts and others in the cytoplasm. We were not able to distinguish which lanthanides were localized in the individual cell structures. However, a previously published study on the localization of individual lanthanides in *D. quadricauda* showed that neodymium and cerium were localized specifically in the chloroplast, while lanthanum and gadolinium were in the cytoplasm [29]. Cerium, neodymium and lanthanum were the most predominant lanthanides found in red mud (Table 1; 35.3, 17.6 and 16.4%, respectively), so the observed bodies should comprise at least one of them. However, it is not clear whether the observed bodies were specific for only one lanthanide or a mixture and more detailed experiments with different single lanthanides (as in [29]) are needed. Lanthanide localization in different cellular compartments (chloroplast and cytoplasm) could play an important role in increasing photosynthetic rate by activation of Rubisco occurring in the stromal compartment of chloroplasts [48], while in the cytoplasm, particularly lanthanum could have a function in stabilization of the cytoskeleton and be involved in some intracellular signaling pathways [49]. Separation of lanthanides from other metals is technologically challenging due to their similar chemical properties. Thus, an understanding of specific compartmentalization of lanthanides in algal cells might be useful for their biotechnological separation by cell fractionation.

### 3.3. Beneficial Effects of Red Mud on Algal Growth

Red mud contains many different elements including some routinely added to algal nutrient media as microelements. Moreover, it also contains other metals necessary for algal growth, such as calcium, magnesium, and iron. The addition of the elements to the growth medium in pure form increases the price of algal cultivation, especially in a scaled up system. Thus, it was of interest to determine whether the red mud could supplement such micronutrients when they are absent from the nutrient medium. Indeed, *D. quadricauda* grew in the red mud even in the absence of added microelements. With increasing red mud concentration there was a noticeably better growth in the presence of red mud over the control cultures. The growth improvement was best evidenced at the highest red mud concentrations and low mean light intensity (Figure 4C). This could be caused by two factors or their combination. Firstly, more metals would be present at high red mud concentrations and they might accumulate more in the cells. Secondly, at low light intensity, the growth of algae is limited and any advantage (e.g., presence of micronutrients) compared to controls will be most evident. Cultures grown in the presence of red mud but the absence of added microelements accumulated more total (Figure 5) and individual lanthanides (Ce, La, Nd) (Figure 6) than cultures grown in complete nutrient medium with added microelements (Figure 5 and Figure 6). This suggests that nutrient limitation promoted accumulation of lanthanides. High levels of accumulation demonstrated in algal cells was previously observed and explained through their ability to form metabolites chelated with compounds [50]. Cultures might also have profited from the presence of red mud compounds, stimulated by bio-absorption of essential metal ions required for microbial metabolic activity and thus achieve higher growth rates than expected [19,40,41].

Given the higher accumulation of lanthanides under conditions of nutrient limitation, their presence might be another possible explanation for growth improvement. Complete nutrient medium contains optimal concentrations of all compounds necessary for growth and reproduction of algae [51]. The only metals not present in nutrient medium, or in the control cells, are the lanthanides. Numerous papers have reported that lanthanides, can stimulate growth and development of plants [52] despite the fact that they are not essential elements. Lanthanides have been demonstrated to exhibit diverse physiological effects on plants and animals [53] due to their involvement in different metabolic pathways, photosynthesis, membrane stability, and stress resistance [54]. They can interact with a great number of biological macromolecules to form stable complexes. Consequently, they were found to act as a substitute for Ca^2+^ ions in some biological functions [55,56]. Although almost all experiments were carried out on higher plants, the substitution of missing calcium by lanthanides was also demonstrated in algae [57]. The feasibility of this hypothesis and identification of affected metabolic processes remains a challenge for future research.

Thus, green algae could be used for bio-mining of lanthanides from red mud as well as for red mud recycling. Algal intracellular accumulation capacity increased with red mud concentration perhaps due to beneficial effects of lanthanides. Interestingly, red mud was able to replace the micronutrients normally added to algal growth medium, thus, decreasing the price of algal cultivation, especially in large scale. Moreover, under micronutrient limitation conditions, lanthanide accumulation further improved. This study provides an initial examination of the bio-mining capacity of green algae. At this point, there are at least two possible downstream applications of algal biomass enriched in lanthanides. The biomass could be directly used as fertilizer [30]. Alternatively, the lanthanides could be isolated from the enriched biomass either chemically or after biomass fractionation, taking into account the information on lanthanide localization to different cell compartments. 

## 4. Materials and Methods 

### 4.1. Microalgal Strains

For these experiments, three model organisms were used: *Desmodesmus quadricauda* (Turpin) Brébisson (previously named as *Scenedesmus quadricauda*, strain Greifswald/15) and *Parachlorella kessleri*, strain 255, were obtained from the Culture Collection of Autotrophic Organisms, Institute of Botany (CCALA, Czech Acad. Sci., Třeboň, Czech Republic), *Chlamydomonas reinhardtii* wild-type strain CC-1690 was obtained from Chlamydomonas Resource Center at (University of Minnesota, St. Paul, MN, USA). All the cultures were obtained sterile and were maintained under sterile conditions both for routine sub-culturing and for the experiments. Cultures of *D. quadricauda* and *P. kessleri* were cultivated in liquid mineral medium as described before (Table 4, upper part) [58,59]. Cultures of *C. reinhardtii* were cultured in modified HS medium (Table 4, middle part) [60]. The cultures were maintained by sub-culturing to appropriate nutrient medium solidified by agar approximately every three weeks and grown at room temperature on a light shelf illuminated by an incident light intensity of 100 µmol/m^2^/s.

### 4.2. Laboratory Experimental Photobioreactor

A set of glass cylinders (inner diameter 36 mm, height 500 mm, volume 300 mL) were placed in a thermostatic bath (30 °C) and continuously illuminated from one side by a panel of dimmable fluorescent lamps (DULUX L55 W/950 Daylight, OSRAM, Milano, Italy) allowing adjustment of the incident light intensity from 16 to 780 µmol/m^2^/s. The cylinders were ‘‘aerated’’ using a mixture of air and CO_2_ (2%, *v/v*) at a flow rate of 15 L/h. The pH of cultures was maintained in the range 6.5–7.5 by the addition of 1 M NaOH. The experiments were carried out in a batch culture regime. 

### 4.3. Measurement of Light Intensity 

For routine light measurement, a quantum/radiometer-photometer (LI-COR, Lincoln, NE, USA) was used. For mean light intensity measurements in the cylinders, an ULM-500 light meter (WALZ, Effeltrich, Germany) equipped with microspherical quantum sensor US-SQS/L was used. To obtain a measure of light energy absorbed by a layer of cell suspension grown at different incident light intensities and different optical densities (concentrations of cells), the mean light intensity (I_m_) was calculated according to the Lambert–Beer formula: I_m_ = (I_i_ I_t_)/ln(I_i_/I_t_), where I_i_ is the incident light intensity measured by the microspherical quantum sensor US-SQS/L positioned in geometrical center of the cultivation cylinder filled with clear nutrient medium and I_t_ is the transmitted light intensity measured at the same position in cultivation cylinder filled either with algal suspension only (control) or by algal suspension supplemented by corresponding amount of red mud suspension (red mud treated cultures).

### 4.4. Preparation of Experimental Cultures 

Cultures were inoculated from plates and cultivated for approximately three days at 30 °C and at a continuous incident light intensity of 500 μmol/m^2^/s. The number of cells in the pre-grown cultures was counted in a Bürker chamber (Meopta, Přerov, Czech Republic) and the cultures for the experiment were diluted to the same initial cell concentration (8 × 10^5^ cells/mL) at the beginning of the experiment and then cultivated in batch culture without any dilution until the end of the experiment (2–5 days). For the experiments, the microalgae were cultivated at a constant temperature (30 °C) and under continuous light of incident light intensity 500 μmol/m^2^/s. 

The composition of incomplete medium lacking added microelements, used for some experiments with *D. quadricauda* was the standard nutrient medium (Table 4, upper part) without addition of microelements (Table 4, bottom part). To prepare cultures limited for microelements, cells of *D. quadricauda* from the pre-grown culture were pelleted and washed twice with medium lacking added microelements (Table 4, upper part) before resuspension to a known cell concentration (8 × 10^5^ cells/mL) in the same medium. Cultures were cultivated in batch culture for 3 days. The pH of the red mud-supplemented nutrient medium (measured by Orion^TM^ Versa Star Pro^TM^ pH meter, ThermoFisher Scientific, Waltham, MA, USA) was in the range from 6.5 to 7.5 at the beginning of cultivation, providing consistent conditions for growth and development of microalgae.

### 4.5. Determination of Cell Number

The number of algal cells was followed by counting cells under transmitted light in a Bürker counting chamber using a BX51 microscope (Olympus, Tokyo, Japan). Values were expressed as the number of cells/mL. 

### 4.6. Estimation of Growth Rates of Cultures

The growth rate **µ** was expressed as a doubling of cell number per day. The growth parameters were calculated according to the formula: N_t_ = N_0_ 2**^µ^** ^t^, from which **µ** = (log_2_ (Nt/N_0_))/t_._ Parameters N_0_ and N_t_ are cell numbers in cultures at the beginning and end of experiment of time interval t; **µ** is the growth rate expressed in doubling/day.

### 4.7. Preparation of Samples for ICP-MS

Cultures were centrifuged (ROTINA 380R, HETTICH, Tuttlingen, Germany) at 4750 rpm for 5 min, washed three times in distilled water and the pellet was frozen at −70 °C and freeze-dried. Samples of algal biomass (0.1 g) were digested with 3 mL of 67% HNO_3_ and 0.5 mL of 30% H_2_O_2_ in a PTFE microwave oven (MLS1200 MEGA, Gemini bv, Apeldoorn, Netherland) at 250–600 W for 20 min. After evaporation of excess acid, the resulting solution was transferred to a volumetric flask supplemented with 0.67% HNO_3_ [57].

### 4.8. Determination of Element Content (ICP-MS) 

For the determination of lanthanide content in the stock solution and in algal biomass, the ICP-MS analytical method was used. ICP-MS measurements were performed using an Elan DRC-e (Perkin Elmer, Concord, ON, Canada) equipped with a concentric PTFE nebuliser, a cyclonic spray chamber, a high-efficiency quartz torch and a dynamic reaction cell (DRC) for the elimination of spectral interference. Distilled and demineralized water (Millipore, Bedford, MA, USA) was used to prepare all solutions. Samples were passed through a 0.45 μm nylon syringe filter and diluted 1:10 using water. Values were expressed as milligram per kilogram (mg/kg) of dry weight [57].

### 4.9. Fluorescence Microscopy

Fluo-4 fluorescent dye was used to display intensification of the fluorescence signal as a consequence of binding to lanthanide cations. A dense algal suspension (1 mL) from control cultures, and from cultures treated with red mud were centrifuged at 5000× *g* for 3 min. The cell pellets were washed with 1 mL of 0.1% sodium dodecyl sulfate, mixed well and centrifuged again at 5000× *g* for 3 min. This washing step was repeated until all possible red mud contaminants were removed (at least three times). The samples were further washed with 1 mL of PBS (phosphate buffered saline), spun at 5000× *g* for 3 min, and the pellet was re-suspended in 1 mL of PBS. In order to stain algal cells, 100 μL of algal suspension, 150 μL of zirconium beads (diameter 0.7 mm), and 100 μL of 2.5 μM Fluo-4 (Molecular Probes, Eugene, OR, USA) freshly diluted in PBS were mixed in 2-mL Eppendorf tubes and vortexed (Vortex Genie2, Scientific Industries, Bohemia, NY, USA) at 4 °C for 1 min at full speed. To complete the staining process, the samples were incubated at room temperature for 2 h while covered with aluminum foil. The samples were then centrifuged at 5000× *g* for 3 min and re-suspended in 100 μL of PBS. To observe the location of the lanthanides, 15 μL of cell suspension was put onto a glass slide and mixed with SlowFade Gold Antifade Reagent (Molecular Probes), preventing the fluorescent dye from fading. For microscopy, a BX51 fluorescence microscope (Olympus) equipped with U-MWIBA2 filter block (excitation 460–490 nm/emission 510–550 nm) was used. Photomicrographs were taken using a DP72 digital camera [29].

### 4.10. Red Mud 

#### 4.10.1. Red Mud Sampling

The red mud used for this study was provided by Envirotis Holding zrt. (Budapest, Hungary) (www.envirotis.hu), a company concerned with reclamation of red mud deposits. Red Mud originated in Depo #7 of the red mud disposal site, located in Almásfüzítő, Hungary, situated on the banks of the river Danube. The coordinates of the sampling site are (N 59°21′33′′; W 26°47′18′′), close to the sonic core drilling #25. The samples were collected from approx. 1–1.2 m depth measured from the red mud surface. At this depth the texture of red mud resembles jelly and wet soil, as opposed to the top surface of the deposit being carbonated due to CO_2_ uptake from the atmosphere. 

#### 4.10.2. Red Mud Treatment 

To determine the effect of red mud on the selected species of microalgae and to verify their ability to take up lanthanides, red mud-supplemented nutrient medium was used for cultivation. The red mud stock solution was prepared by mixing 10% (*w/v*) red mud with de-ionized water and sterilized by autoclaving. The red mud solution was added to the appropriate nutrient medium to a final concentration (*w/v*) of 0.03%, 0.05% and 0.1%, forming a suspension.

## Figures and Tables

**Table 1:** Data on the quality and homogeneity of lanthanides in the red mud from Almásfüzítő, Hungary.

Element	Symbol	Content mg/kg	Avg mg/kg	SD	Avg%
Scandium	Sc	80–110	95	12.2	8.1
Yttrium	Y	95–140	118	18.4	10.1
Lanthanum	La	140–260	200	49.0	17.2
Cerium	Ce	300–550	425	102.1	36.5
Praseodymium	Pr	25–49	37	9.8	3.2
Neodymium	Nd	132–210	171	31.8	14.7
Samarium	Sm	26–38	32	4.9	2.7
Europium	Eu	5–8	7	1.2	0.6
Gadolinium	Gd	20–32	26	4.9	2.2
Dysprosium	Dy	20–35	28	6.1	2.4
Erbium	Er	11–18	15	2.9	1.2
Ytterbium	Yb	11–15	13	1.6	1.1
**Total lanthanides**			**1167**		**100.0**

**Table 2:** Concentration range of the main constituents of red mud.

**Element**	**Symbol**	**Content g/kg**	**Avg g/kg**	**SD**	**Avg%**
Sodium	Na	46.0–49.1	47.6	1.55	17.183
Aluminum	Al	29.6–37.7	33.7	4.05	12.165
Silicon	Si	15.6–32.6	24.1	8.5	8.700
Calcium	Ca	20.3–45.2	32.7	6.2	11.804
Titanium	Ti	19.5–19.6	19.6	0.05	7.057
Manganese	Mn	1.4–1.5	1.45	0.05	0.523
Iron	Fe	146–147.6	147	0.8	53.066
		**mg/kg**	**mg/kg**		
Lithium	Li	70.2–74.7	72.5	2.25	0.026
Beryllium	Be	4.4–4.7	4.6	0.15	0.002
Boron	B	54.6–65.6	60.1	5.5	0.022
Magnesium	Mg	480.5–667.5	574.0	93.5	0.207
Barium	Ba	45.3–52.2	48.7	3.45	0.018
Vanadium	V	741.6–750.7	746.6	4.8	0.270
Chromium	Cr	352.3–370.9	361.5	9.3	0.130
Cobalt	Co	35.3–33.9	34.6	0.7	0.012
Nickel	Ni	173.7–283.5	228.6	54.9	0.083
Copper	Cu	73.1–76.5	74.53	1.7	0.027
Zinc	Zn	112.5–113.4	112.95	0.45	0.041
Gallium	Ga	22.5–22.9	22.7	0.2	0.008
Arsenic	As	98.1–100.8	98.85	1.35	0.036
Rubidium	Rb	1.3–1.3	1.3	0	0.000
Strontium	Sr	584.0–623.2	603.6	19.6	0.218
Zirconum	Zr	445.7–457.2	451.45	5.75	0.163
Niobium	Nb	55.1–55.6	55.35	0.25	0.020
Molybdenum	Mo	11.8–12.1	11.95	0.15	0.004
Palladium	Pd	4.9–5.0	4.95	0.05	0.002
Silver	Ag	1.1–1.2	1.15	0.05	0.000
Cadmium	Cd	1.2–1.4	1.3	0.1	0.000
Tin	Sn	11.3–11.6	11.45	0.15	0.004
Antimony	Sb	11.6–11.7	11.64	0.05	0.004
Tellurium	Te	1.2–1.4	1.3	0.1	0.000
Hafnium	Hf	11.2–11.7	11.45	0.25	0.004
Tantalum	Ta	4.5–4.5	4.5	0	0.002
Wolfram	W	2.9–3.0	2.95	0.05	0.001
**Total elements**			**309.7**	**g/kg**	

**Table 3:** The growth rate (**µ**) of *Desmodesmus quadricauda*, *Chlamydomonas reinhardtii* and *Parachlorella kessleri* at different concentrations of red mud expressed as doubling of number of cells per day.

Concentration of Red Mud	Number of Cells, 10^6^/mL		Growth Rate µ
%	0 h	2 days	
*Desmodesmus quadricauda*			
0	0.8	34.4	2.71
0.03	0.8	30.4	2.62
0.05	0.8	30.5	2.63
0.1	0.8	23.3	2.43
*Chlamydomonas reinhardtii*			
0	0.8	25.75	2.50
0.03	0.8	23.83	2.45
0.05	0.8	20.03	2.32
0.1	0.8	12.63	1.99
*Parachlorella kessleri*			
0	0.8	21.24	2.37
0.03	0.8	20.41	2.34
0.05	0.8	16.25	2.17
0.1	0.8	15.37	2.13

**Table 4:** Composition of nutrient media for all experimental species.

Compound	Weight g/L	MW	Molarity µmol/L	Element
Macroelements for *Desmodesmus quadricauda* and *Parachloreella kessleri*				
KNO_3_	2.021	101.1	19990.1	N
K_2_HPO_4_	0.14	174.2	803.7	P
KH_2_PO_4_	0.34	136.1	2498.2	K
MgSO_4_·7H_2_O	0.988	246.5	4008.1	Mg
CaCl_2_·2H_2_O	0.011	147.0	74.8	Ca
FeNaEDTA	0.018	367.0	49.0	Fe
Macroelements for *Chlamydomonas reinhardtii*				
NH_4_Cl	0.5	53.4	9363.3	N
K_2_HPO_4_	1.44	174.2	8266.4	P
KH_2_PO_4_	0.72	136.1	5290.2	K
MgSO_4_·7H_2_O	1.18	246.5	4787.0	Mg
CaCl_2_·2H_2_O	0.02	147.0	136.1	Ca
FeNaEDTA	0.018	367.0	49.0	Fe
Composition of microelements common for all species				
H_3_BO_3_	0.003	61.8	4.85	B
ZnSO_4_·7H_2_O	0.00143	287.6	4.97	Zn
MnSO_4_·4H_2_O	0.0012	223.0	5.38	Mn
CuSO_4_·5H_2_O	0.00124	231.7	5.35	Cu
CoSO_4_·7H_2_O	0.0014	227.0	6.17	Co
(NH_4_)_6_Mo_7_O_24_·4H_2_O	0.00184	1235.8	1.49	Mo
